# Effects of Transcutaneous Vagus Nerve Stimulation on Cerebral Perfusion and Cognitive Function Under Simulated High‐Altitude Hypobaric Hypoxia

**DOI:** 10.1002/brb3.71729

**Published:** 2026-08-30

**Authors:** Xiaojun Yu, Jinhao Lyu, Jiayu Huang, Yuheng Mao, Wenxia Zhou, Jian Hu, Yongqin Xiong, Xiaoxiao Ma, Jianxing Hu, Caohui Duan, Chenran Wang, Xiangbing Bian, Lin Ma, Jianhui Wang, Xin Lou

**Affiliations:** ^1^ Department of Radiology The First Medical Center of Chinese PLA General Hospital Beijing China; ^2^ State Key Laboratory of National Security Specially Needed Medicines Academy of Military Medical Sciences Beijing China

**Keywords:** arterial spin labeling, cerebral blood flow, cognitive function, hypobaric hypoxia, transcutaneous vagus nerve stimulation

## Abstract

**Background**: The aim of this study is to investigate the effects of hypobaric hypoxia (simulated altitude of 3600 m) on regional cerebral blood flow (CBF) and cognitive function, and to explore the regulatory role of transcutaneous vagus nerve stimulation (tVNS).

**Methods**: Forty‐three healthy Han Chinese males (18–45 years) were enrolled at the Chinese PLA General Hospital (June 2024–December 2025). During 24‐h hypobaric hypoxia exposure via a specialized hypobaric chamber, participants were randomly assigned to tVNS (*n* = 23) or non‐tVNS (*n* = 20) groups. A non‐exposure cohort (*n* = 18) was included to correct for cognitive learning effects. Arterial spin labeling quantified CBF in the anterior, middle, and posterior cerebral artery territories and hippocampus at baseline, exposure, and post‐exposure; the Repeatable Battery for the Assessment of Neuropsychological Status assessed cognitive function at the same time points.

**Results**: Hypoxia‐induced compensatory CBF increases across all examined regions in the control group (*p* < 0.05). In the tVNS group, CBF modulation was region‐specific: frontotemporal CBF was higher at exposure than post‐exposure and higher at post‐exposure than baseline; occipital and hippocampal CBF were higher at exposure than at both other time points, with no difference between post‐exposure and baseline. Only temporal lobe CBF was significantly higher in the tVNS group than the control group (76.22 ± 7.49 vs. 70.88 ± 8.93, *p* = 0.039). Hypoxia impaired visuospatial ability, language, and attention and delayed memory (all *p* < 0.05), but tVNS did not ameliorate these deficits.

**Conclusions**: Simulated 3600 m hypobaric hypoxia induces compensatory regional CBF elevations. tVNS selectively increases temporal lobe perfusion but does not ameliorate cognitive impairment, warranting optimized studies to clarify its neuroprotective role.

## Graphical abstract

This graphical abstract illustrates the study design of this research. Participants were divided into tVNS (n = 23) and control (n = 20) groups. Baseline MRI was acquired before chamber entry. Biochemical and cognitive tests were performed at three phases: pre‐chamber, in‐chamber (with tVNS for the intervention group), and post‐exit. Follow‐up MRI were performed immediately upon exiting the chamber and repeated 24 h after exit. Quantitative CBF maps from MRI were analyzed in predefined brain regions.

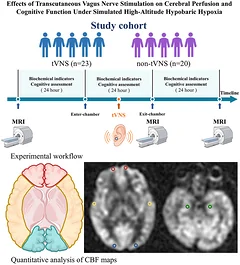

## 1 Introduction

The brain exhibits the highest oxygen consumption per unit mass among all human organs, rendering it extremely sensitive to fluctuations in oxygen demand and partial pressure and thus the most vulnerable tissue to hypoxic stress (Zhu et al. 2022).

As one of the most common and representative hypoxic exposure scenarios, high‐altitude environments are stratified by elevation into three grades: high altitude (1500–3500 m), very high altitude (3500–5500 m), and extreme altitude (>5500 m). With the rise in altitude, the gradual drop in barometric pressure substantially reduces the partial pressure of inspired oxygen, which in turn decreases arterial oxygen saturation and induces persistent relative hypoxia in brain tissue; moreover, the degree of hypoxia deteriorates progressively with increasing elevation (Burtscher et al. 2021; Rickards 2015). Acute mountain sickness (AMS), a syndrome of headache, dizziness, and malaise, can, without timely intervention, progress to life‐threatening high‐altitude cerebral and pulmonary edema (Bartsch and Swenson 2013), highlighting the threat of high‐altitude hypoxia, in which hypoxic stress is the most prominent physiological stressor and the core inducer of related health risks (Howe et al. 2025). To cope with the challenges posed by hypoxic stress, the brain initiates multiple compensatory pathways. Among them, the adaptive regulation of cerebral blood flow (CBF) is a key mechanism for maintaining the stability of cerebral oxygen delivery (CDO_2_), ensuring basic oxygen supply and neuronal survival. Specifically, in lowland individuals exposed to normobaric or hypobaric hypoxia, CBF typically increases compensatorily to maintain the homeostasis of CDO_2_ (Mallat et al. 2022).

Despite the existence of such compensatory mechanisms in the brain, hypoxia may still induce cognitive abnormalities: the core mechanism of high‐altitude‐related cognitive dysfunction is hypoxemia, which impairs multiple cognitive domains by disrupting cerebral oxygenation and neuronal metabolism (Bottura et al. 2025; McMorris et al. 2017). Notably, hypoxia‐related symptoms induced by acute high‐altitude exposure usually abate gradually with the body's acclimatization. Arterial spin labeling (ASL) MRI uses endogenous arterial water as a tracer to noninvasively and quantitatively measure regional CBF with favorable spatial specificity (Troudi Habibi et al. 2025), enabling dynamic monitoring of perfusion changes in hypobaric hypoxic environments. Vagus nerve stimulation (VNS) is a neuromodulatory technique applied in the treatment of various neurological disorders via electrical stimulation of the vagus nerve (Sharma et al. 2025). Transcutaneous vagus nerve stimulation (tVNS), a non‐invasive neuromodulation technique, has garnered rising attention in research due to its excellent safety and straightforward operational procedures (Arsava et al. 2022; Batson et al. 2022; Feygina et al. 2023). Existing studies have verified that tVNS amplifies task‐triggered CBF elevation while concurrently modulating cortical neural excitability and systemic autonomic homeostasis (Jung et al. 2024; Kunii et al. 2021; Ma et al. 2019). Mounting evidence shows non‐invasive VNS produces neuroprotection via autonomic modulation, elevated cerebral perfusion, and enhanced neuroplasticity. As a typical form of VNS, tVNS modulates the balance between sympathetic and parasympathetic biomarkers during stimulation. The robust coupling between these autonomic physiological readouts and stress responses substantiates tVNS's capacity to stabilize autonomic equilibrium in healthy populations (Drost et al. 2025). Beyond peripheral autonomic tuning, tVNS engages central arousal circuits centered on the locus coeruleus–norepinephrine system to ameliorate cognitive processing and improve working memory capacity (Tan et al. 2024). However, the regulatory effects of VNS on CBF in cortical regions supplied by the anterior, middle, and posterior cerebral arteries, as well as in the hippocampus, and its specific impact on cognitive function under hypobaric hypoxia remain poorly understood.

Accordingly, the present study utilized a hypobaric hypoxia chamber to simulate an altitude of 3600 m combined with tVNS intervention. ASL was applied to quantify CBF in the cortical territories of the anterior cerebral artery (ACA), middle cerebral artery (MCA), and posterior cerebral artery (PCA), as well as in the hippocampus, at the axial basal ganglia plane. We further dynamically observed temporal changes in regional perfusion and cognitive function across different time points. This study aimed to characterize the effects of hypobaric hypoxia on perfusion across distinct brain regions, explore the potential interventional benefits of tVNS, and thereby provide experimental evidence and feasible strategies for cerebral protection in populations working at high altitudes.

## 2 Materials and Methods

All participants provided written informed consent before enrollment. This study was conducted in strict accordance with the ethical principles outlined in the Declaration of Helsinki. The research protocol was reviewed and approved by the Ethics Committee of the Chinese PLA General Hospital (approval No.: S2024‐175‐01).

### 2.1 Study Patients

**FIGURE 1:**
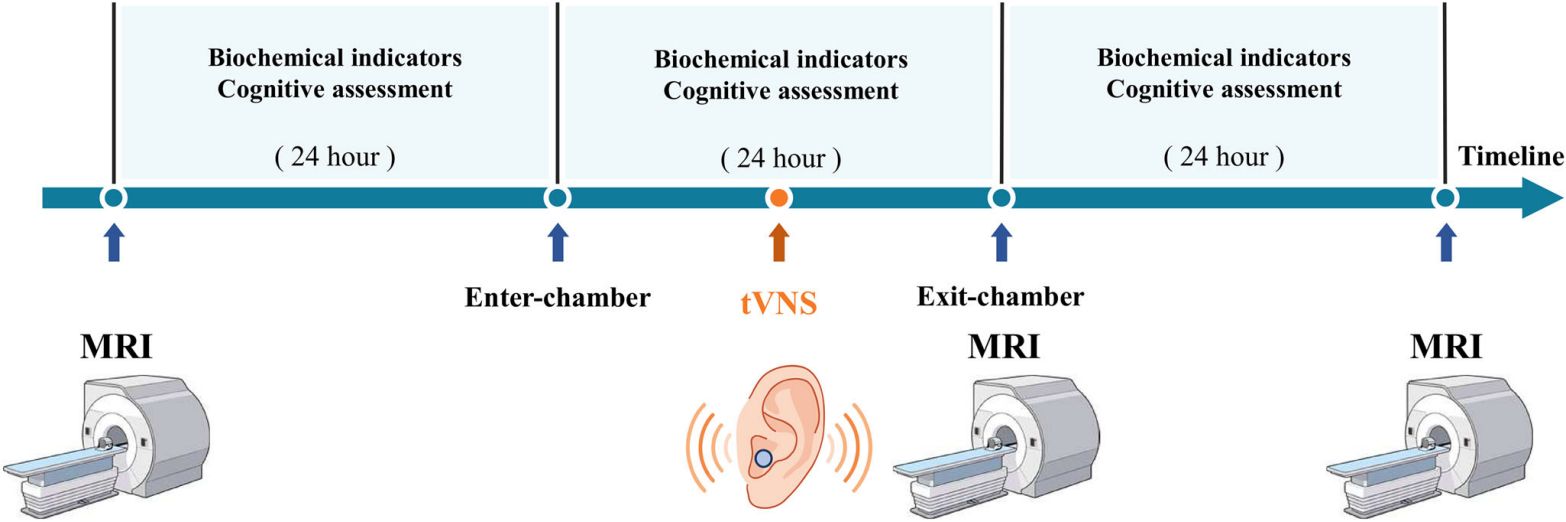
The overall experimental procedure and workflow. tVNS, transcutaneous vagus nerve stimulation.

This study enrolled healthy adult male participants of Han Chinese ethnicity, aged 18–45 years, with at least a high school education, between June 2024 and December 2025 at the Chinese PLA General Hospital. All participants were in good physical health and had no contraindications for MRI, no abnormal laboratory test results or MRI findings, and no recent travel or residence in high‐altitude regions (defined as areas with an altitude of 2500 m or higher) within the past month. All subjects were continuously exposed for 24 h in the DYC‐3285 hypobaric hypoxic chamber to simulate the plateau environment at an altitude of 3600 m. The internal temperature of the chamber was maintained at 25°C ± 3°C DYC‐3285 hypobaric hypoxic chamber to simulate 64.9 kPa and a partial pressure of oxygen of around 13.6 kPa. Stimulation parameters: The stimulation site was the inner cavum conchae of the left auricle. One cycle lasted 4 min, with a total of four cycles (16 min in total). Intratrain pulse frequency = 25 Hz; pulse duration = 0.2 ms. During hypoxic exposure, participants were randomized to receive active auricular tVNS or sham stimulation. Electrodes were placed and fixed identically on the left auricle in both groups; the sham group received identical electrode placement and fixation with no electrical output throughout the intervention. MRI scans and cognitive assessments were conducted at baseline (pre‐exposure), immediately upon exiting the chamber (exposure), and 24 h post‐exposure. Exclusion criteria were (a) failure to complete 24 h of exposure in the chamber; (b) missing imaging or cognitive assessment data at any of the three time points (baseline, immediate exit from chamber, or 24 h post‐exposure); and (c) severe image artifacts. The overall experimental workflow is illustrated in Figure 1.

### 2.2 Urinalysis and Glycemic Markers

Urinalysis and glycemic markers were measured in all subjects at three time points: pre‐exposure (baseline), 24 h during hypobaric hypoxic exposure (in‐chamber), and 24 h after termination of exposure (post‐chamber). These markers were used to evaluate systemic physiological responses to hypobaric hypoxia and tVNS intervention.

### 2.3 MRI Parameters

MR images were acquired using a 3.0‐T MR scanner with a 32‐channel head coil. Three‐dimensional pseudo‐continuous arterial spin labeling was acquired with the following parameters: repetition time (TR) = 5025 ms, post‐labeling delay = 2.0 s, slice thickness = 4.0 mm, 36 axial slices, field of view = 240 mm × 240 mm, and total scan duration of 4 min 23 s.

### 2.4 ASL‐Derived CBF Quantification

**FIGURE 2:**
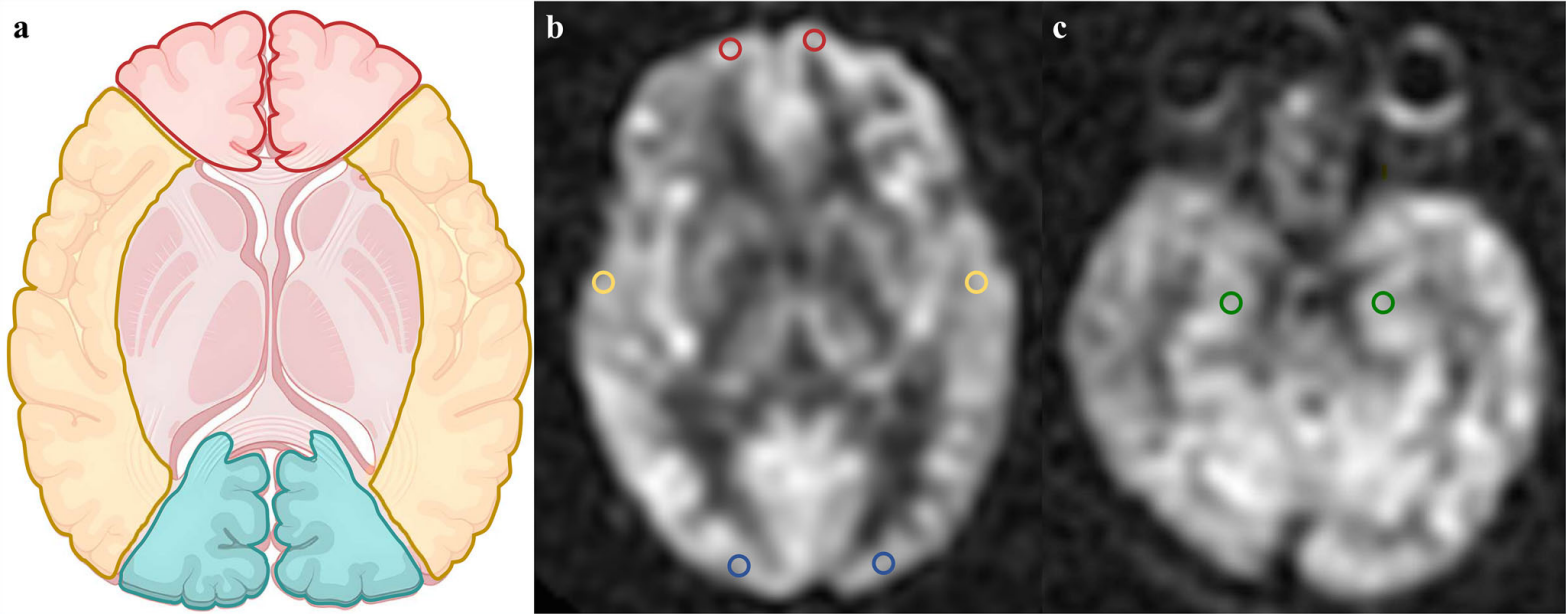
Schematic of cerebral vascular territories and ROI placement on CBF maps. (a) Axial diagram at basal ganglia level: ACA (red), MCA (yellow), and PCA (blue) territories. (b) Axial CBF map at the same level: circular ROIs in bilateral cortical regions of ACA (red), MCA (yellow), and PCA (blue). (c) Axial CBF map at hippocampal level: bilateral hippocampal ROIs (green circles) for regional CBF measurement. CBF, cerebral blood flow; ROI, region of interest; ACA, anterior cerebral artery; MCA, middle cerebral artery; PCA, posterior cerebral artery.

All MR images were reviewed by two independent observers with 7 and 11 years of radiology imaging experience, who were blinded to the group assignment (tVNS vs. non‐tVNS). The mean of the observers’ measurements was used as the final value for each quantitative parameter to minimize interobserver bias. Axial CBF maps acquired at the basal ganglia level were used for quantitative analysis. Based on the vascular territories of the ACA, MCA, and PCA (Figure 2a), circular regions of interest (ROIs) with a minimum area of 30 mm^2^ were manually delineated on the CBF maps. ROIs were placed in the bilateral cortical regions perfused by the ACA, MCA, and PCA (Figure 2b), as well as in the bilateral hippocampal regions (Figure 2c), to measure regional CBF values. Quantification of CBF was performed using the ASL perfusion analysis module on the GE AW4.6 workstation. Perfusion difference maps were generated by subtracting the control images from the paired labeled images. Tissue and blood T1 values, labeling efficiency, slice thickness, and other relevant parameters were imported into the standard pCASL quantification formula to calculate absolute CBF values (unit: mL/100 g/min).

### 2.5 Cognitive Assessment

Cognitive performance was quantified using an adapted version of the Repeatable Battery for the Assessment of Neuropsychological Status (RBANS), a validated instrument that assesses five core cognitive domains: (1) immediate memory, (2) visuospatial/constructional ability, (3) language proficiency, (4) attention capacity, and (5) delayed memory. The 12‐subtest battery required 20–30 min for administration, with raw scores subsequently converted into age‐adjusted index scores (higher values indicating superior cognitive function). Cognitive assessments were administered to each participant at three time points: pre‐exposure, 24 h during hypobaric hypoxic exposure, and 24 h after termination of exposure. To counteract the learning effect of repeated cognitive testing, an additional cohort of healthy subjects who did not undergo simulated 3600 m hypobaric hypoxic exposure was enrolled as a non‐exposure control cohort. This cohort underwent identical cognitive assessments at two time points, allowing for quantification and correction of the bias introduced by repeated testing.

### 2.6 Statistical Analysis

Statistical analyses were performed using SPSS (version 27.0; IBM Corp). The concordance of quantitative parameters between observers was examined using intraclass correlation coefficient (ICC) analysis. One‐way analysis of variance was used for normally distributed continuous variables, and the Kruskal–Wallis test for non‐normally distributed variables, to compare measurements across the three time points (baseline, exposure, and post‐exposure) within each group, as well as across the three groups (non‐exposure, non‐tVNS, and tVNS) at each time point. The Mann–Whitney U test or *t*‐test was used to compare continuous variables between two groups. Data following a normal distribution are expressed as mean ± standard deviation, while non‐normally distributed data are expressed as median (first and third quartiles). Statistical significance was set at *p* < 0.05.

## 3 Results

### 3.1 Participant Characteristics

A total of 43 healthy subjects were enrolled in this study. All subjects completed 24‐h exposure in a hypobaric hypoxic chamber simulating an altitude of 3600 m and were randomly assigned to two groups: the tVNS group (*n* = 23) and the non‐tVNS group (*n* = 20). In addition, an additional cohort of 18 healthy subjects without exposure to 3600 m altitude was enrolled to control for the learning effect of cognitive testing. The median age of the tVNS group and the non‐tVNS group was 25.0 (23.0–29.0) and 23.0 (20.5–27.5) years, respectively, with no statistically significant difference between groups (*p* > 0.05).

### 3.2 Urinary Parameters

In the non‐tVNS group, statistically significant differences were detected in urine specific gravity (SG) between exposure and post‐exposure time points, as well as in urine pH between baseline and exposure time points (*p* < 0.05) (Table 1). By contrast, no significant differences were observed in either parameter across all time points in the tVNS group (Table 2).

**TABLE 1:** Changes in urine SG, urine pH, and HbA1c levels across different time points in the non‐tVNS group.

Indicator	Baseline	Exposure	Post‐exposure	*p* Value
Urine SG	1.026(1.022–1.030)	1.029(1.025–1.032)	1.024(1.016–1.027)	0.026 (0.369^a^, 0.753^b^, 0.021^c^)
Urine PH	6.00(5.50–6.50)	6.25(6.00–7.38)	6.25(6.00–7.00)	0.039 (0.017^a^, 0.051^b^, 0.658^c^)
HbA1c	4.20 ± 0.75	4.20 ± 0.69	4.13 ± 0.74	0.936 (0.983^a^, 0.746^b^, 0.763^c^)

^a^, ^b^, and ^c^ represent the *p*‐values compared by baseline and exposure, baseline and post‐exposure, and exposure and post‐exposure, respectively.

Abbreviations: SG, specific gravity; tVNS, transcutaneous vagus nerve stimulation; HbA1c, glycosylated hemoglobin, Type A1C.

**TABLE 2:** Changes in urine SG, urine pH, and HbA1c levels across different time points in the tVNS group.

Indicator	Baseline	Exposure	Post‐exposure	*p* Value
Urine SG	1.025 ± 0.006	1.025 ± 0.007	1.020 ± 0.007	0.154 (0.137^a^, 0.737^b^, 0.070^c^)
Urine PH	6.00(6.00–6.50)	6.50(6.00–7.00)	6.50(6.00–6.50)	0.218 (0.109^a^, 0.698^b^, 0.180^c^)
HbA1c	4.67 ± 0.83	4.49 ± 0.62	4.45 ± 0.65	0.542 (0.395^a^, 0.301^b^, 0.852^c^)

^a^, ^b^, and ^c^ represent the *p*‐values compared by baseline and exposure, baseline and post‐exposure, and exposure and post‐exposure, respectively.

Abbreviations: SG, specific gravity; tVNS, transcutaneous vagus nerve stimulation; HbA1c, glycosylated hemoglobin, Type A1C.

### 3.3 ASL‐Derived CBF Parameters

**FIGURE 3:**
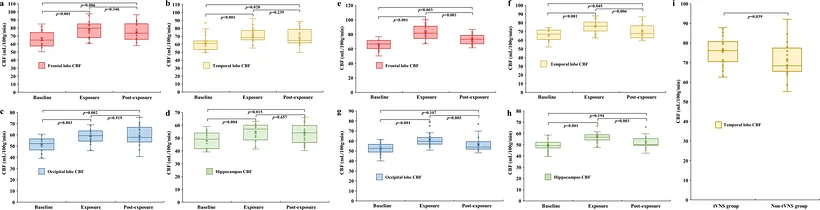
Box plots illustrating CBF distributions in the frontal lobe, temporal lobe, occipital lobe, and hippocampus across three time points (baseline, exposure, and post‐exposure) in the non‐tVNS and tVNS groups. Panels (a–d) depict longitudinal CBF changes in the frontal lobe, temporal lobe, occipital lobe, and hippocampus of the non‐tVNS group at baseline, exposure, and post‐exposure stages. Panels (e–h) show the corresponding longitudinal CBF variations in the four brain regions of the tVNS group. (i) Comparative analysis of temporal lobe CBF between the non‐tVNS and tVNS groups during the exposure stage. CBF, cerebral blood flow; tVNS, transcutaneous vagus nerve stimulation.

The interobserver ICCs for the CBF parameters ranged from 0.86 to 0.91, confirming good interobserver reliability. In the non‐tVNS group, significant longitudinal changes in CBF across baseline, exposure, and post‐exposure time points were detected in the frontal lobe, temporal lobe, occipital lobe, and hippocampus (all *p* < 0.05), with corresponding *p* values of 0.001, 0.003, 0.003, and 0.008, respectively. Post hoc pairwise comparisons demonstrated that CBF values in these brain regions were significantly higher at both exposure and post‐exposure time points than at baseline, whereas no significant differences were found between exposure and post‐exposure measurements (Table 3 and Figure 3a–d). In the tVNS group, CBF exhibited significant longitudinal variations across baseline, exposure, and post‐exposure time points within the frontal lobe, temporal lobe, occipital lobe, and hippocampus (all *p* < 0.001). Further pairwise comparisons revealed distinct patterns: CBF values in the frontal and temporal lobes were higher during exposure than at post‐exposure time points and higher at post‐exposure than baseline. For the occipital lobe and hippocampus, CBF values were significantly higher at exposure than both baseline and post‐exposure time points, whereas no significant difference was observed between post‐exposure and baseline (Table 4 and Figure 3e–h). Between‐group comparisons showed no significant differences in CBF values of the frontal lobe, occipital lobe, and hippocampus between the tVNS group and non‐tVNS group at baseline, exposure, or post‐exposure time points (*p* > 0.05). Only temporal lobe CBF differed significantly between the two groups at the exposure time point (76.22 ± 7.49 vs. 70.88 ± 8.93, *p* = 0.039, 95% confidence interval [CI]: 0.29–10.40) (Figure 3i), whereas no significant between‐group differences were found at baseline or post‐exposure, indicating that tVNS exerts a selective regulatory effect on temporal lobe perfusion during hypobaric hypoxia.

**TABLE 3:** CBF in different brain regions across time points in the non‐tVNS group.

Parameters	Baseline	Exposure	Post‐exposure	*p* Value
Frontal lobe CBF	65.61 ± 9.50	77.95 ± 10.27	74.89 ± 10.80	0.001 (<0.001^a^, 0.006^b^, 0.346^c^)
Temporal lobe CBF	61.05(53.28–64.15)	68.40(65.58–77.40)	64.70(61.75–78.78)	0.003 (0.001^a^, 0.028^b^, 0.239^c^)
Occipital lobe CBF	51.68 ± 6.49	58.94 ± 6.57	59.18 ± 8.94	0.003 (0.003^a^, 0.002^b^, 0.919^c^)
Hippocampus CBF	49.15(41.75–53.88)	57.00(48.50–60.03)	54.15(46.53–59.90)	0.008 (0.004^a^, 0.015^b^, 0.657^c^)

^a^, ^b^, and ^c^ represent the *p*‐values compared by baseline and exposure, baseline and post‐exposure, and exposure and post‐exposure, respectively.

Abbreviations: tVNS, transcutaneous vagus nerve stimulation; CBF, cerebral blood flow.

**TABLE 4:** CBF in different brain regions across time points in the tVNS group.

Parameters	Baseline	Exposure	Post‐exposure	*p* Value
Frontal lobe CBF	65.68 ± 6.60	82.34 ± 10.04	72.88 ± 6.95	<0.001 (<0.001^a^, 0.003^b^, <0.001^c^)
Temporal lobe CBF	65.74 ± 5.95	76.22 ± 7.49	70.12 ± 8.14	<0.001 (<0.001^a^, 0.045^b^, 0.006^c^)
Occipital lobe CBF	52.60(48.90–56.60)	59.60(56.80–63.60)	53.90(51.80–59.30)	<0.001 (<0.001^a^, 0.107^b^, 0.005^c^)
Hippocampus CBF	49.20(47.10–52.20)	56.70(54.20–58.90)	50.00(49.50–55.40)	<0.001 (<0.001^a^, 0.194^b^, 0.003^c^)

^a^, ^b^, and ^c^ represent the *p* values compared by baseline and exposure, baseline and post‐exposure, and exposure and post‐exposure, respectively.

Abbreviations: tVNS, transcutaneous vagus nerve stimulation; CBF, cerebral blood flow.

### 3.4 Cognitive Performance

Within both the tVNS and non‐tVNS groups, statistically significant differences in immediate memory, attention capacity, and delayed memory were observed across baseline, exposure, and post‐exposure time points (all *p* < 0.05). Further pairwise comparisons demonstrated that scores for these three domains were higher at post‐exposure than at exposure and higher at exposure than at baseline. In contrast, no statistically significant differences were observed in visuospatial/constructional ability and language proficiency across the three time points within each group (Supplementary Tables S1 and S2). Repeated cognitive assessments in the non‐exposure control cohort revealed that post‐test performance on all five cognitive domains (immediate memory, visuospatial/constructional ability, language proficiency, attention capacity, and delayed memory) was significantly higher in the second assessment compared with the first (all *p* < 0.05), confirming the presence of a learning effect associated with repeated testing (Supplementary Table S3).

**FIGURE 4:**
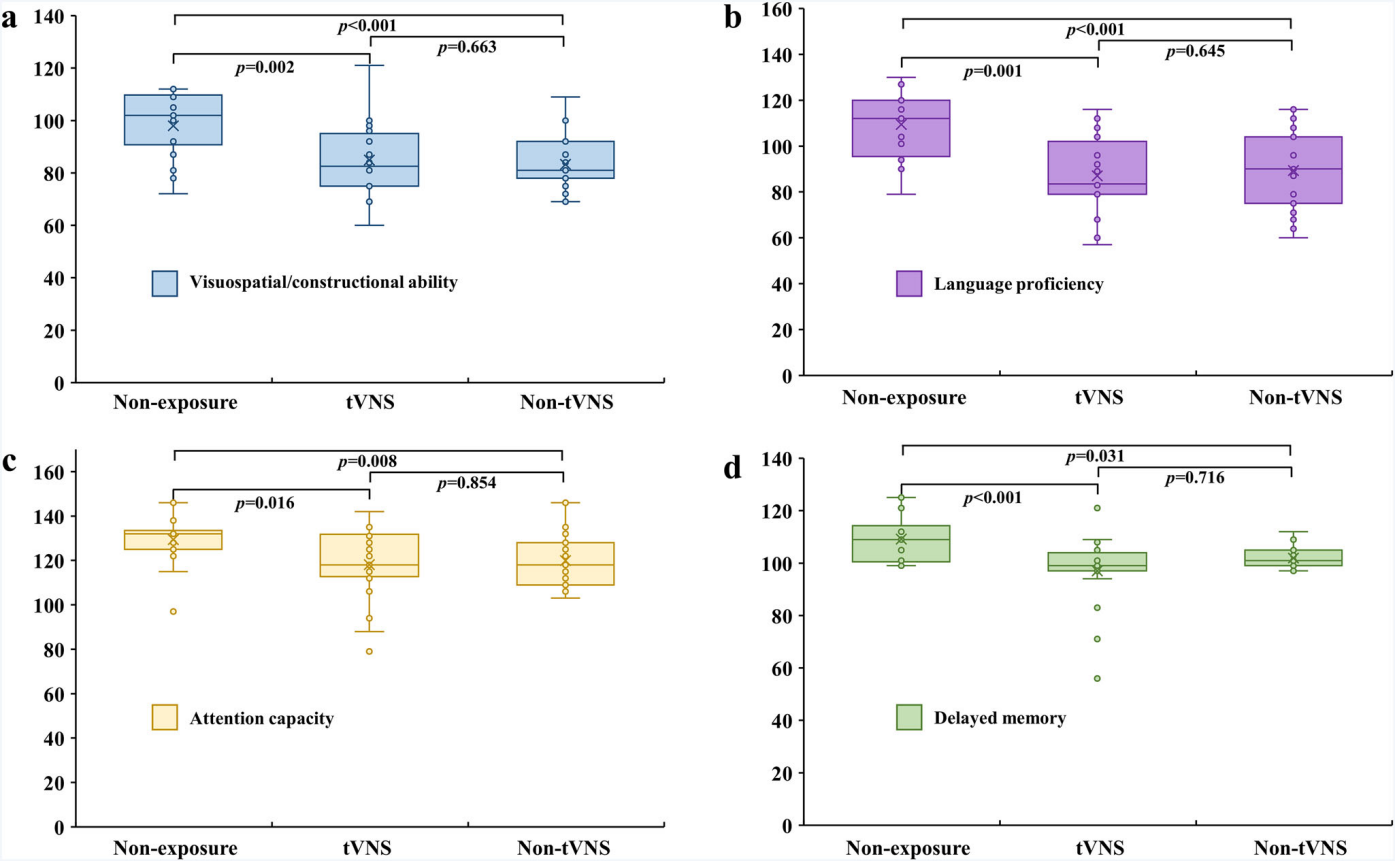
Box plots illustrating intergroup differences in visuospatial/constructional ability, language proficiency, attention capacity, and delayed memory among the non‐exposure group and two hypoxic cohorts (tVNS and non‐tVNS groups). Panels (a–d) compare (a) visuospatial/constructional ability, (b) language proficiency, (c) attention capacity, and (d) delayed memory across the control, tVNS, and non‐tVNS groups. tVNS, transcutaneous vagus nerve stimulation.

Further comparative analyses of baseline cognitive performance between the non‐exposure group and the two exposure groups (tVNS and non‐tVNS) demonstrated no statistically significant differences among the three groups in any of the five cognitive domains (*p* > 0.05) (Supplementary Table S4). Intergroup comparisons were further performed to contrast cognitive outcomes at the exposure time point for the two hypoxic cohorts against the second assessment of the non‐exposure group (second assessment, corrected for learning effect). Significant intergroup differences were detected in visuospatial/constructional ability (*p* = 0.001), language proficiency (*p* < 0.001), attention capacity (*p* = 0.015), and delayed memory (*p* = 0.001) (Table 5 and Figure 4a–d).

**TABLE 5:** Intergroup comparisons of cognitive function scores: the non‐exposure group (second assessment, adjusted for learning effect) versus the tVNS and non‐tVNS hypoxic cohorts at exposure time point.

Indicator	Non‐exposure (*n* = 18)	Non‐tVNS (*n* = 20)	tVNS (*n* = 23)	*p* Value
Immediate memory	127.89 ± 16.16	116.10 ± 19.52	118.48 ± 17.85	0.112 (0.048^a^, 0.101^b^, 0.666^c^)
Visuospatial ability	98.00 ± 12.41	84.90 ± 14.35	83.26 ± 9.93	0.001 (0.002^a^, <0.001^b^, 0.663^c^)
Language proficiency	109.44 ± 14.44	87.00 ± 16.73	89.30 ± 17.21	<0.001 (0.001^a^, <0.001^b^, 0.645^c^)
Attention capacity	132.00(125.00–133.50)	118.00(112.75–131.75)	118.00(109.00–128.00)	0.015 (0.016^a^, 0.008^b^, 0.854^c^)
Delayed memory	109.00(100.50–114.25)	99.00(97.00–104.00)	101.00(99.00–105.00)	0.001 (<0.001^a^, 0.031^b^, 0.716^c^)

^a^, ^b^, and ^c^ represent the *p* values compared by non‐exposure and non‐tVNS, non‐exposure and tVNS, and non‐tVNS and tVNS, respectively.

Abbreviation: tVNS, transcutaneous vagus nerve stimulation.

## 4 Discussion

The present study utilized a hypobaric hypoxic chamber to simulate a 3600 m high‐altitude environment and employed ASL‐MRI combined with tVNS intervention to systematically investigate the effects of hypobaric hypoxic exposure on regional CBF in the ACA, MCA, and PCA territories, as well as the hippocampus. We further explored the regulatory role of tVNS in this context and analyzed the characteristics of cognitive function changes under hypoxic conditions, aiming to provide experimental evidence and theoretical support for the protection of brain function in high‐altitude working populations.

Consistent with prior studies, our results demonstrated that CBF was significantly increased in subjects after exposure to a hypobaric hypoxic environment compared with baseline (Kunii et al. 2021). This global elevation in CBF under acute hypoxia suggests that the body initiates compensatory cerebrovascular vasodilation to counteract inadequate ambient oxygen availability, thereby maintaining CDO_2_ and meeting the basal metabolic demands of the brain (Harris et al. 2013; Li et al. 2020; Pollock et al. 2008). Similarly, ASL‐based investigations under high‐altitude conditions have reported increased regional CBF, particularly within the frontal and sensorimotor cortices, key regions responsible for cognitive function and motor control (Pagani et al. 2011), further supporting the role of CBF elevation as a critical adaptive response to acute hypoxia.

Acute hypoxia at 3500 m elicits an immediate increase in CBF, which gradually normalizes with prolonged exposure as physiological acclimatization proceeds (Turner et al. 2024). Over time, ventilatory and hematological compensatory mechanisms, including hyperventilation and elevated hemoglobin concentrations, act synergistically to preserve systemic oxygen delivery and maintain tissue oxygenation stability (Wang et al. 2018). In the present study, we extended these findings by demonstrating that the regional CBF of the temporal lobe (a core MCA‐perfused region) was significantly higher in the tVNS group than the control group under hypobaric hypoxia. This effect was particularly prominent in brain regions implicated in sensory processing and cognitive function, implying that these areas are more vulnerable to hypoxic stress. Notably, tVNS effectively augmented regional perfusion to sustain neural activity in these regions, which is consistent with prior evidence that tVNS amplifies task‐induced increases in CBF (Kunii et al. 2021).

By contrast, prior work has reported that mild hypoxia encountered by pilots flying at high altitudes without supplemental oxygen correlates with reduced CBF in the right temporal, occipital, and cerebellar regions (Liu et al. 2021). These regions are critical for sensory integration, visuospatial processing, and motor coordination; hence, even subclinical regional hypoperfusion may precipitate slowed reaction time, impaired judgment, and subsequent risks to flight safety. Our observation that tVNS selectively elevates temporal lobe CBF under hypobaric hypoxia carries evident translational implications. The temporal lobe governs memory encoding and multimodal sensory processing, which are highly susceptible to hypoxic injury. Notably, this perfusion‐regulating effect is not limited to high‐altitude hypoxic conditions; it offers novel insights for reperfusion interventions in acute ischemic stroke, wherein vascular recanalization and restoration of regional cerebral perfusion constitute the core therapeutic targets. Existing studies have verified that tVNS can safely modulate inflammatory responses in patients with acute ischemic stroke caused by large‐vessel anterior circulation occlusion (Laurido‐Soto et al. 2025). Furthermore, additional evidence indicates that this intervention facilitates upper extremity motor function recovery in patients with subacute ischemic stroke, with favorable tolerance and no remarkable adverse reactions (Wu et al. 2020). Nevertheless, translational discussion should objectively distinguish the pathological injury pathways between high‐altitude hypoxia and ischemic stroke, and the therapeutic targets of the two conditions should not be equated. Although tVNS ameliorated hypoxia‐induced temporal hypoperfusion, no significant intergroup differences in cognitive function improvement were observed. Therefore, alterations in CBF alone cannot sufficiently establish that tVNS exerts a comprehensive neuroprotective effect.

With respect to urinary parameters, the control group displayed significant alterations in urine SG during and after hypobaric hypoxia exposure, along with pronounced shifts in urine pH during exposure relative to baseline. These observations indicate that hypobaric hypoxia disrupts water–electrolyte metabolism and acid–base equilibrium, which is consistent with the known physiological effects of acute hypoxia on renal function. Conversely, no such significant deviations were detected in the tVNS group, suggesting that tVNS may preserve internal environmental homeostasis via modulation of autonomic nervous function (Austelle et al. 2024; Fang et al. 2023). This finding highlights an additional mechanism by which tVNS may exert protective effects during hypobaric hypoxia, beyond its modulation of cerebral perfusion.

Cognitive function in this study was evaluated using an adapted version of the RBANS. While both groups exhibited a trend toward higher immediate memory and delayed memory scores during hypobaric hypoxia compared with baseline, this trend may be attributed to the learning effect of repeated testing, as evidenced by the improved performance in the non‐exposure control cohort. To mitigate this confounding influence, we compared cognitive performance between the hypobaric hypoxia exposure groups and the non‐exposure control group (corrected for the learning effect). The results demonstrated that the hypoxia‐exposed groups performed significantly worse across multiple cognitive domains, including visuospatial/constructional ability, language proficiency, attention, and delayed memory, which aligns with previous research confirming that acute high‐altitude exposure impairs complex cognitive function and exerts a detrimental effect on general cognitive performance (Dykiert et al. 2010; Wang et al. 2022). Given the hippocampus's critical role in explicit memory, particularly spatial learning and memory, functional or structural disturbances in this region may directly contribute to the observed cognitive impairments (Schacter 2025; Slotnick 2024).

Nevertheless, at altitudes ranging from 2300 to 5700 m, enhanced lactate production may also exert adaptive effects: lactate can act as an alternative neuronal energy substrate, promote vasodilation, and activate cellular signaling pathways that facilitate hypoxic acclimatization (Proia et al. 2016). This adaptive mechanism may partially counteract the detrimental effects of hypoxia on cerebral function, potentially explaining why some cognitive domains (e.g., immediate memory) did not show overt impairment in the within‐group comparisons. Furthermore, mood states, especially tension and fatigue, are closely correlated with the severity of AMS symptoms (Bottura et al. 2025). Cognitive disturbances such as diminished memory and attention, which govern higher order functions including thought organization, task prioritization, and decision‐making, are also commonly observed under hypoxic conditions (Turner et al. 2015). These findings are reinforced by a recent hypobaric chamber study simulating altitudes of 3000–4050 m, in which subjects with AMS symptoms displayed reduced vigor and increased fatigue and depression (Figueiredo et al. 2022), underscoring the close link between hypoxia, mood, and cognitive function.

Our study has some limitations. First, participants could distinguish active tVNS from sham stimulation due to the absence of cutaneous sensory feedback in the sham group, which may introduce expectation bias. Hence, expectancy effects and cutaneous sensory input cannot be completely ruled out as confounding factors accounting for the observed temporal‐lobe CBF changes. Second, only healthy adult males were enrolled as study subjects with a limited sample size, which may restrict the generalizability and universal applicability of the study findings to a certain extent. Third, the intervention duration was only 24 h; the long‐term effects of tVNS were not investigated, and key parameters such as stimulation intensity and frequency were not optimized. Future studies may extend the intervention period and refine stimulation protocols to further systematically elucidate the regulatory patterns and temporal effects of tVNS. Fourth, this study only simulated an altitude of 3600 m and did not explore the interventional effects of tVNS under extreme hypoxic conditions at higher altitudes (e.g., above 5000 m). Subsequent research may expand the altitude gradient to systematically improve investigations into the neuroprotective effects of tVNS on brain function at different levels of hypoxic exposure.

In conclusion, our findings demonstrate that simulated hypobaric hypoxia at 3600 m induces compensatory increases in regional CBF across multiple brain regions. Although tVNS selectively modulates temporal lobe CBF during hypobaric hypoxia, it fails to significantly ameliorate cognitive impairment in this setting. Further investigations with optimized study designs including larger sample sizes, extended intervention durations, refined tVNS parameters, and expanded altitude gradients are needed to clarify the neuroprotective mechanisms of tVNS and to establish more targeted interventions for cerebral protection among high‐altitude working populations.

## Author Contributions

**Xiaojun Yu**: writing – review & editing, writing – original draft, resources, investigation, conceptualization, formal analysis, methodology, visualization, data curation. **Jinhao Lyu**: writing – review & editing, writing – original draft, resources, investigation, conceptualization, formal analysis, methodology, visualization, data curation. **Jiayu Huang**: writing – review & editing, investigation, methodology, data curation. **Yuheng Mao**: writing – review & editing, investigation, methodology, data curation. **Wenxia Zhou**: writing – review & editing, investigation, methodology, formal analysis. **Jian Hu**: writing – review & editing, investigation, methodology, data curation. **Yongqin Xiong**: writing – review & editing, formal analysis, methodology, data curation. **Xiaoxiao Ma**: Writing – review & editing, Formal analysis, Visualization. **Jianxing Hu**: Writing – review & editing, Investigation, Data curation. **Caohui Duan**: Writing – review & editing, Formal analysis, Visualization. **Chenran Wang**: Writing – review & editing, Visualization, Data curation. **Xiangbing Bian**: Writing – review & editing, Resources. **Lin Ma**: Writing – review & editing, Supervision. **Jianhui Wang**: Writing – review & editing, Supervision, Conceptualization. **Xin Lou**: Writing – review & editing, Supervision, Conceptualization.

## Funding

The authors have nothing to report.

## Conflicts of Interest

The authors declare no conflicts of interest.

## Supporting information




**Supporting File 1**: brb371729‐sup‐0001‐SuppMat.docx

## Data Availability

Data available upon request due to privacy and ethical restrictions.
