# Supplementary material for: Effects of Transcutaneous Vagus Nerve Stimulation on Cerebral Perfusion and Cognitive Function Under Simulated High‐Altitude Hypobaric Hypoxia
Source: Brain Behav. 2026 Aug 30;16(9):e71729. doi: 10.1002/brb3.71729 (PMC13527389; doi:10.1002/brb3.71729)
Supplement: Supplementary file 1 [file BRB3-16-e71729-s001.docx]

**Supplement Table 1**. Changes in cognitive function scores across baseline, exposure, and post-exposure time points in non-tVNS group.

| **Indicator** | Baseline | Exposure | Post-exposure | *P* value |
| --- | --- | --- | --- | --- |
| Immediate  memory | 84.90 ± 13.29 | 116.10 ± 19.52 | 131.60 ± 13.82 | <0.001  (<0.001^*^, <0.001^#^, 0.003^&^) |
| Visuospatial  ability | 84.00(73.50–99.00) | 82.50(75.00–95.00) | 84.00(73.00–90.75) | 0.935  (0.714^*^, 0.796^#^, 0.968^&^) |
| Language  proficiency | 102.50(87.50–104.00) | 83.50(79.00–102.00) | 90.00(90.00–103.25) | 0.207  (0.142^*^, 0.653^#^, 0.118^&^) |
| Attention  capacity | 115.60 ± 11.51 | 118.15 ± 16.18 | 126.65 ± 13.03 | 0.035  (0.559^*^, 0.014^#^, 0.055^&^) |
| Delayed  memory | 94.00(88.75–97.00) | 99.00(97.00–104.00) | 103.00(99.50–108.00) | <0.001  (0.008^*^, <0.001^#^, 0.122^&^) |

*, #, & represent the *p* values compared by baseline and exposure, baseline and post-exposure, and exposure and post-exposure, respectively.

tVNS, transcutaneous vagus nerve stimulation

**Supplement Table 2**. Changes in cognitive function scores across baseline, exposure, and post-exposure time points in tVNS group.

| **Indicator** | Baseline | Exposure | Post-exposure | *P* value |
| --- | --- | --- | --- | --- |
| Immediate  memory | 84.39 ± 15.13 | 118.48 ± 17.85 | 130.13 ± 18.11 | <0.001  (<0.001^*^, <0.001^#^, 0.024^&^) |
| Visuospatial  ability | 86.65 ± 12.85 | 83.26 ± 9.93 | 81.61 ± 11.83 | 0.329  (0.325^*^, 0.145^#^, 0.631^&^) |
| Language  proficiency | 89.57 ± 17.36 | 89.30 ± 17.21 | 93.43 ± 16.02 | 0.651  (0.958^*^, 0.440^#^, 0.409^&^) |
| Attention  capacity | 117.57 ± 9.67 | 119.91 ± 12.60 | 126.17 ± 9.17 | 0.022  (0.455^*^, 0.008^#^, 0.049^&^) |
| Delayed  memory | 94.00(81.00–97.00) | 101.00(99.00–105.00) | 105.00(99.00–109.00) | <0.001  (<0.001^*^, <0.001^#^, 0.360^&^) |

*, #, & represent the *p* values compared by baseline and exposure, baseline and post-exposure, and exposure and post-exposure, respectively.

tVNS, transcutaneous vagus nerve stimulation

**Supplement Table 3.** Comparison of cognitive function scores between the first and second assessments in the non-exposure group.

| **Indicator** | **First assessment** | **Second assessment** | ***P* value** |
| --- | --- | --- | --- |
| Immediate memory | 84.56 ± 4.68 | 127.89 ± 16.16 | <0.001 |
| Visuospatial ability | 80.67 ± 10.28 | 98.00 ± 12.41 | <0.001 |
| Language proficiency | 95.28 ± 12.75 | 109.44 ± 14.44 | 0.004 |
| Attention capacity | 112.00(109.00–128.00) | 132.00(125.00–133.50) | 0.001 |
| Delayed memory | 97.00(92.50–99.00) | 109.00(100.50–114.25) | <0.001 |

**Supplement Table 4**. Baseline cognitive function scores across the non-exposure, non-tVNS, and tVNS groups.

| **Indicator** | Non-exposure (n = 18) | Non-tVNS (n = 20) | tVNS (n = 23) | *P* value |
| --- | --- | --- | --- | --- |
| Immediate memory | 84.56 ± 4.68 | 84.90 ± 13.29 | 84.39 ± 15.13 | 0.991  (0.932^*^, 0.966^#^, 0.893^&^) |
| Visuospatial ability | 80.67 ± 10.28 | 85.80 ± 16.34 | 86.65 ± 12.85 | 0.335  (0.245^*^, 0.163^#^, 0.837^&^) |
| Language proficiency | 95.00(86.00–104.00) | 102.50(87.50–104.00) | 92.00(79.00–101.00) | 0.352  (0.606^*^, 0.428^#^, 0.146^&^) |
| Attention capacity | 112.00(109.00–128.00) | 118.00(106.00–125.00) | 115.00(112.00–125.00) | 0.940  (0.871^*^, 0.682^#^, 0.844^&^) |
| Delayed memory | 97.00(92.50–99.00) | 94.00(88.75–97.00) | 94.00(81.00–97.00) | 0.118  (0.075^*^, 0.065^#^, 0.931^&^) |

*, #, & represent the *p* values compared by non-exposure and non-tVNS, non-exposure and tVNS, and non-tVNS and tVNS, respectively.

tVNS, transcutaneous vagus nerve stimulation
